# Neurosurgical Intervention for Nerve and Muscle Biopsies

**DOI:** 10.3390/diagnostics14111169

**Published:** 2024-05-31

**Authors:** Ali A. Mohamed, Thomas Caussat, Edwin Mouhawasse, Rifa Ali, Phillip M. Johansen, Brandon Lucke-Wold

**Affiliations:** 1 Charles E. Schmidt College of Medicine, Florida Atlantic University, Boca Raton, FL 33431, USA; 2 College of Medicine, University of Central Florida, Orlando, FL 32827, USA; 3 Department of Neurosurgery and Brain Repair, University of South Florida, Tampa, FL 33613, USA; 4 Department of Neurosurgery, University of Florida, Gainesville, FL 32608, USA

**Keywords:** nerve biopsy, neurosurgical biopsy, neuropathy, neuropathology, technique

## Abstract

(1) Background: Neurologic and musculoskeletal diseases represent a considerable portion of the underlying etiologies responsible for the widely prevalent symptoms of pain, weakness, numbness, and paresthesia. Because of the subjective and often nonspecific nature of these symptoms, different diagnostic modalities have been explored and utilized. (2) Methods: Literature review. (3) Results: Nerve and muscle biopsy remains the gold standard for diagnosing many of the responsible neurological and musculoskeletal conditions. However, the need for invasive tissue sampling is diminishing as more investigations explore alternative diagnostic modalities. Because of this, it is important to explore the current role of neurosurgical intervention for nerve and muscle biopsies and its current relevance in the diagnostic landscape of neurological and musculoskeletal disorders. With consideration of the role of nerve and muscle biopsy, it is also important to explore innovations and emerging techniques for conducting these procedures. This review explores the indications and emerging techniques for neurological intervention for nerve and muscle biopsies. (4) Conclusions: The role of neurosurgical intervention for nerve and muscle biopsy remains relevant in diagnosing many neurological and musculoskeletal disorders. Biopsy is especially relevant as a supportive point of evidence for diagnosis in atypical cases. Additionally, emerging techniques have been explored to guide diagnostics and biopsy, conduct less invasive biopsies, and reduce risks of worsening neurologic function and other symptoms secondary to biopsy.

## 1. Introduction

Pain, weakness, and sensory deficits, including numbness and paresthesia, are widely prevalent. Chronic pain affects 20.9% of all adults in the United States, [1] reduced muscle strength is reported by 18% of those 60 years and above and 53% of those 80 and above, [2] and peripheral neuropathy, a common cause of numbness and paresthesia, affects over 14.5 million Americans [3]. Symptoms may be due to a wide variety of etiologies, with the broadest classification schemes dividing causes into central or peripheral pathologies, and with further differentiation between neurologic and musculoskeletal disease.

Because symptoms are relatively subjective and difficult to objectively characterize, a variety of diagnostic modalities are used to identify a diagnosis, including physical examination, imaging, and tissue sampling. While electromyography and nerve conduction studies have improved the ability to diagnose such conditions, muscle and nerve biopsies remain the gold standard for diagnosis in many neurologic and musculoskeletal conditions. However, as further studies into the molecular and genetic etiologies of such neurologic and musculoskeletal pathologies are performed, the need for invasive tissue sampling continues to diminish. We aim to discuss this controversy and highlight the role of neurosurgical intervention in nerve and muscle biopsies.

Muscle biopsies are typically performed later during the diagnostic workup, e.g., to confirm the presence of a muscular dystrophy or an inflammatory myositis. However, when the differential diagnosis is extensive, early muscle biopsy can quickly lead to a diagnosis and allow for earlier intervention. Muscle biopsies are performed by a surgeon, commonly a neurosurgeon, in conjunction with a pathologist. Typically, an affected muscle is located by physical examination, although electrodiagnostic or magnetic resonance imaging (MRI) may also be used [4]. Muscle biopsies are often performed under local anesthesia. The surgeon dissects down to the muscle and identifies a group of muscle fibers at the belly of the muscle, after which the surgeon excises in parallel to the length of the muscle fiber a sample approximately 1 cm in length and 0.5 cm in diameter. Once removed, the specimen is packaged for examination by a pathologist. Muscle biopsy typically produces no residual deficits [4]. 

Nerve biopsies are comparable in that they are typically performed later in the diagnostic workup and are also performed by a surgeon, commonly a neurosurgeon, in conjunction with a pathologist. Nerve biopsies are especially useful in diagnosing inflammatory neuropathies, autoimmune neuropathies (e.g., nerve vasculitides and chronic inflammatory demyelinating polyneuropathy), and hereditary neuropathies and can alter treatment in up to 60% of cases [5]. Sensory nerves are the ideal targets rather than motor nerves, as nerve biopsies often result in temporary nerve damage, and sensory deficits are tolerated better than motor weakness. As such, transient sensory loss, which is rarely permanent and infrequently associated with painful paresthesia, may occur after a nerve biopsy. The sural nerve is the most common site of biopsy, although many other nerves may also be sampled when indicated by physical examination, electrophysiological studies, or MRI [5]. Like muscle biopsies, nerve biopsies are commonly performed under local anesthesia, although they require a large incision to ensure safe and adequate dissection from all surrounding fat and connective tissue. After careful dissection, the nerve is isolated and extracted before undergoing extensive preparation for pathologist examination.

## 2. Nerve Biopsy Indications

Nerve biopsy plays an important diagnostic and evaluative role in cases of clear and unclear pathology [6]. Nerve biopsy can also assist in attaining a definitive diagnosis in cases of vasculitis, neurosarcoidosis, neurolymphomatosis, amyloidosis, and neuritic leprosy [7,8,9,10,11]. In addition, nerve biopsy can be instrumental in cases of rapidly progressive peripheral neuropathy or peripheral neuropathy of an unexplained cause [12,13,14,15]. However, the diagnostic role of nerve biopsy may differ from one pathology to another (Table 1) [6]. Despite its variable importance, the many indications and clear diagnostic role make neurosurgical intervention for nerve biopsies an important aspect of the clinical decision making process. 

### 2.1. Nerve Biopsy: High Importance

#### 2.1.1. Vasculitis

Vasculitic neuropathy is a common indication for nerve biopsy with a reported sensitivity of 54.76% and reports of a 5.1% increase in diagnostic yield when undergoing a combined muscle biopsy [17,18,19]. When undergoing a combined muscle and skin biopsy, cutaneous vasculitis was identified in 20% of suspected patients with vasculitic neuropathy [20]. Besides evidence of the potential diagnostic benefits of a full thickness skin biopsy, different quantification strategies have been explored to increase sensitivity and specificity for diagnosing and differentiating vasculitic neuropathies [20,21,22,23]. Current diagnostic recommendations by the Peripheral Nerve Society dictate biopsy for the definitive diagnosis of vasculitic neuropathy and non-systemic vasculitic neuropathy, with the exception of cases of proven systemic vasculitis or diabetic radiculoplexus neuropathy [11,24].

The clinical presentation of vasculitic neuropathy is described as multifocal sensorimotor pure sensory, acute/subacute, painful, asymmetric neuropathy [11]. Systemic vasculitic neuropathy involves small arteries and large arterioles, whereas non-systemic vasculitic neuropathy more typically involves the small arterioles, venules, and capillaries [16].

**Figure 1:**
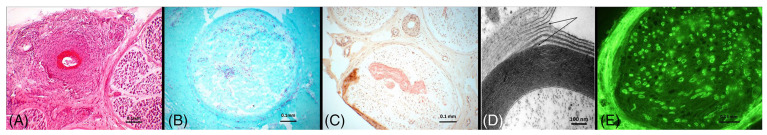
(**A**) Vasculitic neuropathy. Intense vascular and perivascular inflammation of an epineurial blood vessel accompanied by fibrinoid necrosis is noted. (**B**) Leprous neuropathy. Positive Ziehl–Neelsen staining for acid fast bacilli is seen. (**C**) Amyloid neuropathy. Amyloid deposits are evident with Congo red staining. (**D**) IgM paraproteinemic neuropathy. Widening of the myelin outer lamella (arrows) is a characteristic finding. (**E**) MAG neuropathy. Immunofluorescence demonstrates MAG antibodies bound to myelin [6] © 2021 The Authors. Muscle & Nerve published by Wiley Periodicals LLC.

A diagnosis is made upon histologic confirmation of both inflammation within the vessel wall and vascular damage. Other histological findings including predominant axonal changes, perivascular inflammation, hemosiderin deposition, multifocal or asymmetric nerve fiber degeneration, neurovascularization, predominant axonal change, or prominent axonal degeneration, and vascular deposition of IgM, fibrinogen, or complement can support a diagnosis of vasculitic neuropathy (Figure 1) [6,11,16,25].

#### 2.1.2. Neurolymphomatosis

Neurolymphomatosis is another neurological manifestation but is that of neoplastic nerve infiltration by a hematological malignancy [7]. A nerve biopsy for the diagnosis of neurolymphomatosis does not demonstrate 100% sensitivity, but a reported sensitivity of 88% because of the inconsistent distribution of malignant cells [26]. Biopsy is not need for diagnosis of neurolymphomatosis in cases of a known hematological malignancy presenting with symptoms of neurological manifestations. However, neuropathy experienced in cases of latent or hematological malignancies in remission may only be attributed to malignancy through a nerve biopsy [25]. Utilizing the polymerase chain reaction of a biopsy-attained lymphoid infiltrate is an additional diagnostic angle utilized to differentiate between inflammatory and malignant infiltrates through the assessment of clonality [27]. 

Neurolymphomatosis presents with peripheral or cranial neuropathy, plexopathy, and radiculopathy associated with rapidly evolving, asymmetrically distributing, severe pain [7]. A positive nerve biopsy will demonstrate malignant cells, with a non-Hodgkin lymphoma representing the usual underlying hematological malignancy [28].

#### 2.1.3. Peripheral Nerve Tumors

Benign peripheral nerve tumors, commonly schwannomas, neurofibromas, perineurioma, and ganglioneuroma, and malignant peripheral nerve tumors arising de novo or from benign tumors, are another indication for nerve biopsy [29,30,31,32]. The presentation is that of peripheral neuropathy. Perineuriomas may also present clinically as plexopathy but typically present clinically as mononeuropathy [33].

Diagnosis typically requires the identification of characteristic histological findings of the underlying disease with the use of the immunohistochemical stains S100, the glial fibrillary acidic protein (GFAP), the epithelial membrane antigen (EMA), and cluster differentiation 34 (CD34) [29,30,31,32].

#### 2.1.4. Pseudoneoplastic Peripheral Nerve Tumors (Pseudotumors)

Pseudotumors are an important indication for nerve biopsy to attain a definitive diagnosis and differentiate the underlying pathology from a malignancy. Inflammatory pseudotumors and neuromuscular choristomas are specific types of pseudotumors within this rare symptomatic disease category [34].

The typical presentation is progressive, painful mononeuropathy with associated sensory and strength loss. A nerve biopsy of an inflammatory pseudotumor typically reveals chronic inflammatory infiltrates, increased vascularity and lipocytes, and interstitial fibrosis [34,35]. A nerve biopsy of neuromuscular choristomas usually demonstrates well-differentiated muscle fibers, typically skeletal muscle fibers, interspersed among nerve fascicles.

#### 2.1.5. Neuritic Leprosy

Pure neuritic leprosy is a subtype of leprosy that represents up to 8% of all leprosy cases [9]. Nerve biopsy is the diagnostic gold standard, with a reported sensitivity of 33.3–75.9% of cases [36,37,38]. Pure neuritic leprosy presents as an isolated peripheral neuropathy. Definitive diagnosis following a confirmatory biopsy typically demonstrates acid fast bacilli for lepromatous leprosy and epithelioid caseating and noncaseating granulomas for tuberculoid leprosy. Acid fast bacilli are typically found within Schwan cells and foam cells (Figure 1) [9].

### 2.2. Nerve Biopsy: Medium Importance

#### 2.2.1. Amyloidosis

Amyloid neuropathy is a neurological complication of amyloidosis, mainly caused by the deposition of light chain or mutant transthyretin amyloid fibrils [39]. A nerve biopsy for amyloidosis has a sensitivity range from 30 to 100%. A sural nerve biopsy specifically demonstrates a sensitivity of 80% in detecting mutant transthyretin amyloid fibrils [40]. The definitive diagnosis criteria of light chain amyloidosis consist of histopathologic findings of amyloid with supporting evidence of amyloid protein composition [41]. The definitive diagnosis of mutant transthyretin amyloidosis requires evidence of amyloid deposits from a biopsy, as well as a minimum of two symptoms associated with amyloidosis [10]. Although many other test options exist for the conclusive diagnosis of amyloidosis, mainly bone marrow testing and abdominal fat pad biopsy because of their sensitivity, nerve biopsy can provide a definitive diagnosis in cases of negative findings [42,43]. 

Amyloid neuropathy may present with motor symptoms but mainly presents as a sensory neuropathy with autonomic symptoms [44,45]. Variability in symptoms and presentation can be attributed to the focal nature of the disease. Because of this, serial sections are examined to establish the diagnosis. Expected findings are unmyelinated or small myelinated nerve fiber loss. Hematoxylin and eosin or Congo red staining may reveal endo- and epineuria connective tissue deposition of amyloid. Endo- and epineural thickening of blood vessels may also be seen (Figure 1) [46].

#### 2.2.2. Neurosarcoidosis

Neurosarcoidosis is a neurological manifestation that can be attributed to neuropathy and other neurological symptoms experienced by up to 16% of patients diagnosed with sarcoidosis [47,48]. A definitive diagnosis requires a nerve biopsy if clinical manifestations and imaging findings are suggestive of neurosarcoidosis [49]. If extraneural sarcoidosis is evident in a biopsy in the absence of positive nerve biopsy findings, a diagnosis of probable neurosarcoidis may be made when the clinical presentation and imaging findings are supportive [8,48,50]. 

The neurological manifestations of sarcoidosis include cranial, optic, and peripheral neuropathy, endocrine and hypothalamic dysfunction, seizures, meningitis, myelopathy, and myopathy [16,19,24,47,48,49,50]. A confirmatory nerve biopsy demonstrates non-caseating granulomas, typically found in the epi- and perineurium, that are independent of other etiologies such as leprosy and tuberculosis [49].

#### 2.2.3. IgG4-Related Perineural Disease

IgG4-related perineural disease is a rare disease caused by IgG4-positive plasma cell infiltration of tissue within the peripheral nervous system. A definitive diagnosis requires nerve biopsy confirmation of IgG4-positive plasma cell infiltratation [51]. Patients may present with multifocal neuropathy and reported biopsy findings have been variable [52,53,54]. Axonal and myelin degeneration, epineural infiltration of plasma cells, eosinophils, lymphocytes, and myelinated nerve fiber reduction all represent a spectrum of features reported for different patient cases [52,53,55,56]. 

#### 2.2.4. Paraneoplastic Syndromes

Paraneoplastic syndromes (PNSs) can manifest as complex neurological syndromes that can affect multiple parts of the nervous system, including the CNS, neuromuscular junction (NMJ), and peripheral nervous system [57]. They are caused by malignancies which are often occult and thus found as a result of clinical symptoms attributed to the syndrome [58]. Paraneoplastic syndromes affect around 8% of individuals with cancer but are most common in small-cell lung cancer, where around 5% of patients are affected by PNSs, most commonly Lambert–Eaton myasthenic syndrome.

Clinical presentations vary with each neurologic syndrome but may include cognitive and personality changes, cranial nerve deficits, ataxia, paresthesia, weakness, or numbness [58]. Common PNSs include limbic encephalitis and paraneoplastic cellular degeneration within the CNS, Lambert–Eaton myasthenic syndrome and myasthenia gravis within the NMJ, and autonomic neuropathy and subacute sensory neuropathy within the peripheral nervous system [59]. The pathogenesis of these syndromes is poorly understood, but current research supports evidence of the cross-reactivity of nervous system antigens that are also ectopically produced by the tumor, producing an immunological response. Thus, paraneoplastic antibodies, or onconeural antibodies, can develop against target antigens [57]. Additionally, diagnosis is strengthened by the presence of onconeural antibodies in the CSF or serum, which can be highly specific for tumors that may be in earlier stages and not otherwise detected [58]. However, around 30% of patients with PNS may not have detectable antibodies in the serum or CSF. Thus, diagnostic methods focus on imaging (CT/MRI/PET) to determine the source of malignancy, and serologies, EEG, nerve conduction studies, electromyography, as well as CSF analysis for inflammation. 

PNS becomes a more “definite” or “probable” diagnosis when patients demonstrate clinical presentations, the presence of cancer, and positive onconeural antibodies [58]. Current diagnostic procedures do not often include a nerve biopsy to aid diagnosis, as most syndromes rely on nerve conduction or EMG when symptoms of weakness and neuropathy are involved and can be evaluated with less invasive techniques. However, in a case study in 2020, a sural nerve biopsy demonstrating lymphocytic microvasculitis (with both B and T cells) was crucial for a patient presenting with progressive upper and lower limb weakness. The biopsy, along with positive antibodies (anti-Hu and anti-CV2), led to suspicion towards paraneoplastic syndrome, and subsequent testing including a lymph node biopsy helped diagnose small cell lung cancer confined to a solitary lymph node as the source of malignancy [60]. Thus, nerve biopsies in the setting of unclear etiologies of peripheral neuropathies may be beneficial in steering the diagnosis of paraneoplastic syndromes with unique presentations and previous negative testing. 

### 2.3. Nerve Biopsy: Low Importance

#### 2.3.1. Chronic Inflammatory Demyelinating Polyneuropathy (CIDP)

CIDP presents with monophasic, progressive, or relapsing symmetrical sensory and motor deficits, both proximally and distally [61,62]. Although nerve biopsies can be utilized in cases of patients presenting with limited features of demyelination, the diagnosis of CIDP is less reliant on nerve biopsy because of the easy access and availability of supporting criteria from other tests. Investigations have demonstrated that amongst patients diagnosed with CIDP, a nerve biopsy may be utilized as the supportive criteria in only 3% of patients [63]. The main use of nerve biopsy in the context of CIDP is for atypical presentations, when patients are treatment-resistant, or to rule out other etiologies of the presenting symptoms [64].

#### 2.3.2. Paraproteinemic Neuropathy

Paraproteinemic neuropathy is a relatively large class of neuropathy-causing disorders that are less reliant on nerve biopsy. Nerve biopsy in paraproteinemic neuropathies is mainly used to support a causal relationship between the presence of specific paraproteins and the clinical presentation of neuropathy. Typical identification of pathologic paraproteins, and their distinguishment from coincidental non-disease-causing paraproteins, is conducted through the visualization of paraproteins binding to nerve components using indirect immunofluorescence. In addition, high titers of IgM antibodies against ganglioside Q1b and myelin-associated glycoprotein alleviate the need for nerve biopsy to determine the cause of the presenting demyelinating neuropathy [6,65].

A nerve biopsy can assist in specific diagnosis confirmation of cryoglobulinemic neuropathy. A nerve biopsy in these cases will typically reveal large fiber axonal degeneration. Concomitant features of demyelination, vasculopathy, and vasculitis are also common [66]. Amorphous material can also be visualized within vacuoles in myelin sheaths [66,67].

A nerve biopsy can also assist in confirming paraproteinemic neuropathy in cases where IgM antibodies are visualized against neural antigens including GM1, GM2, GD1a, and GD1b. A nerve biopsy can also be utilized to assist in the confirmatory diagnosis of cases where IgG or IgA antibodies are present, visualization of complement binding to myelin or widely spaced myelin, or visualization of immunoglobulin binding to myelin (Figure 1)**.** Nerve biopsy features can be specific to different types of paraproteinemic neuropathy [68,69,70,71,72,73,74,75,76,77]. However, because these disorders are less reliant on biopsy for diagnosis, specificity is rarely utilized clinically for diagnosis and confirmation [6,65].

#### 2.3.3. Adult Polyglucosan Body Disease

Adult polyglucosan body disease is typically diagnosed by genetic testing, followed by glycogen branching enzyme activity assays [78]. Nerve biopsy is only indicated if enzyme activity assay results are ambiguous. Reported biopsy findings included polyglucosan bodies; however, these findings were also found in patients without the disease [6,79,80].

#### 2.3.4. Lysosomal and Peroxisomal Storage Disorders

Nerve biopsy is only indicated in the presence of atypical manifestations/presentations or if usual testing fails to determine a highly suspected diagnosis of neuropathy secondary to lysosomal and peroxisomal storage disorders [81]. Nerve biopsy may reveal Zebra or Tuff stone bodies, a result of intralysosomal material accumulation in Schwan cells and neurons, onion bulb structures in patients with Refsum’s disease, or prismatic inclusions in patients with leukodystrophies and other sphingolipidoses [6,81].

#### 2.3.5. Pure Motor Neuropathy

Pure motor neuropathy can be difficult to differentiate from progressive muscular atrophy. In such cases, a nerve biopsy can assist in diagnosis. Nerve biopsy of pure motor neuropathy has demonstrated 95% sensitivity when a biopsy is conducted on the motor branch of the obturator nerve [82]. Biopsy findings suggestive of pure motor neuropathy include high regenerative activity, findings suggestive of demyelination and remyelination, and deposits of axonal inclusions or amyloid. Low regenerative activity and axonal degeneration are more consistent with motor neuron disease and not pure motor neuropathy [6,82].

#### 2.3.6. Diabetic Neuropathy

Nerve biopsy in cases of suspected diabetic neuropathy can be indicated in the presence of atypical manifestations/presentations or to determine the true etiology of the patient’s symptoms when other superimposed disorders, such as an ischemic inflammatory process, may be occurring [83]. Typical cases of diabetic neuropathy do not require morphological confirmation [44].

#### 2.3.7. Cryptogenic Neuropathy

Similarly, nerve biopsy in cases of cryptogenic neuropathy is not typically indicated but may be useful in cases of high suspicion where extensive workup is inconclusive [13,15]. Nerve biopsy findings are expected to yield 0–37% of useful diagnostic information [84,85].

#### 2.3.8. Hereditary Neuropathy

Owing to next generation sequencing, nerve biopsy does not play an important diagnostic role in patients with hereditary neuropathies. Specific select cases may benefit from nerve biopsy, but other diagnostic tools are sufficient in most cases [86].

#### 2.3.9. Other Neuropathies

**Figure 2:**
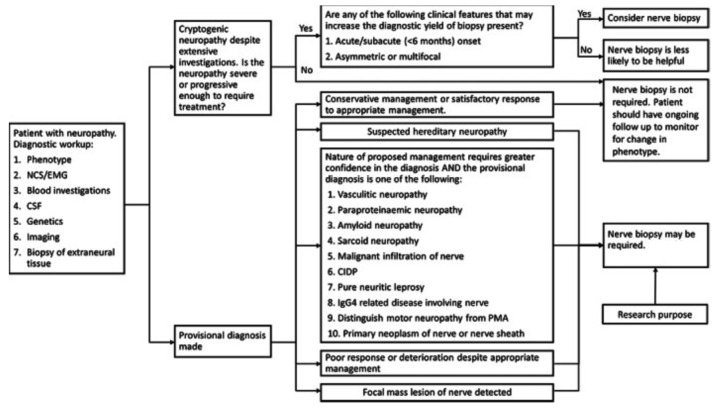
Proposed decision tree to facilitate decision making for nerve biopsy. EMG, electromyogram; NCS, nerve conduction studies [6] © 2021 The Authors. Muscle & Nerve published by Wiley Periodicals LLC.

Consistent with what has been discussed for all nerve biopsy indications of low importance, several other neuropathies that are not reliant on nerve biopsy for diagnosis may still benefit from nerve biopsy in select cases. This includes toxic neuropathies from specific medications, neuropathy secondary to environmental or industrial exposure, diffuse infiltrative lymphocytosis syndrome, and chronic idiopathic sensory axonal neuropathy [87,88,89,90,91,92,93]. Nerve biopsy in such cases may assist in diagnostic clarification in cases of rapid progression or atypical presentations, or in the exploration of disease progression and potential treatments [94,95,96]. Figure 2 demonstrates a proposed decision tree for performing nerve biopsies and Table 2 demonstrates when a biopsy is indicated for a suspected diagnosis.

## 3. Muscle Biopsy Indications

Muscle biopsy is an important component in disease evaluation and diagnosis [97]. A muscle biopsy is specifically required to definitively diagnose hereditary disorders such as muscular and myotonic dystrophies, congenital myopathies, channelopathies, primary metabolic disorders, disorders of carbohydrate and lipid metabolism, and acquired myopathies such as inflammatory, toxic/drug-induced, endocrine, and systemic illness-associated myopathies [98]. Muscle biopsy also plays a significant role in the general evaluation of patients with neuromuscular disease, the progression and course of a disease, and the differentiation of neurogenic and myogenic disorders. Considering the many indications and its large evaluative and diagnostic role, neurosurgical intervention for muscle biopsies remains an important component of medical reasoning and decision making.

### 3.1. Muscle Biopsy: High Indication

#### 3.1.1. Polyarteritis Nodosa (PAN)

Polyarteritis nodosa (PAN) is a medium-sized vessel, immune complex-related vasculitis that causes necrosis [99]. PAN can be caused by viral infections like Hepatitis B, Hepatitis C, HIV, CMV, and Parvovirus B19. However, the majority of cases are idiopathic. This condition affects multiple systems in the body, with central nervous system involvement being associated with higher mortality. A PAN diagnosis can be made following histological confirmation of a muscle, skin, or nerve biopsy of the affected area [100]. 

PAN can affect blood vessels systemically, with the potential of hemorrhage or ischemia in any organ because of the abundance of blood vessels throughout the body [101]. Clinically, PAN can manifest with systemic, cutaneous, neurological, gastrointestinal, urologic, ophthalmologic, cardiac, and respiratory symptoms. The most common neurological manifestations are mononeuritis multiplex occurring in 38–72% of cases, peripheral neuropathy occurring in 74% of cases, central nervous system involvement occurring in 2–28% of cases, and cranial nerve palsy in <2% of cases [100].

Combined muscle and nerve biopsies were effective in providing histologic confirmation of vasculitis in 83% of cases, whereas muscle biopsies alone were only effective at confirming vasculitis in 65% of cases. Histopathology typically demonstrates inflammatory infiltrates: lymphocytes, macrophages, neutrophils, and eosinophils. Granulomas and giant cells are not observed, but fibrinoid necrosis is typically seen in active lesions. Early in the disease course, fibrinoid necrosis will present with neutrophil involvement, followed by lymphocyte and macrophage involvement. In advanced lesions, neoangiogenesis and vascular remodeling will be seen with intimal hyperplasia and diffuse fibrotic changes in the vessel wall. Thrombosis can typically appear, as well as microaneurysms due to severe vessel wall injury [100].

#### 3.1.2. Dystrophinopathy

Dystrophinopathies are X-linked muscle diseases that result in muscle atrophy and fibrosis. The underlying pathophysiology of dystrophinopathies involves a deficiency in the dystrophin protein [102]. The most common dystrophinopathies are Duchenne and Becker muscular dystrophies. Duchenne’s muscular dystrophy (DMD) is recognized by an absence of dystrophin and the dystrophin–glycoprotein complex. This deficiency results in membrane fragility, excessive permeability, improper calcium homeostasis, and oxidative damage. The combined impact of these deficiencies results in muscle cell necrosis [103]. Becker’s muscular dystrophy is a less severe disease, categorized by nonfunctional or decreased production of dystrophin. Because the underlying pathology is less severe, muscular atrophy and other clinical features progress modestly [104]. Although genetic testing has grown in popularity for its ability to detect 95% of pathogenic variants of DMD, muscular biopsy remains as a reliable diagnostic adjunct to genetic testing. A 2018 study of the diagnosis of DMD found that the number of muscular biopsies for DMD has remained at a stable level since 1997. They also found that muscle biopsy can provide enhanced diagnostic capacity for patients with later symptom onset, comorbidities, or a previous normal DMD genetic test [105]. Furthermore, muscle biopsies provide a better understanding of the disease phenotype through quantification of the dystrophin within the tissue.

Duchenne and Becker muscular dystrophies present relatively similarly, mainly distinguished by the timing of symptom presentation. Duchenne typically presents between the ages of 1.2 and 8 years, while Becker typically presents between the ages of 5 and 60 years [105]. Short stature is common, but developmental milestones are achieved at a normal or slightly delayed pace prior to symptom onset. Infants commonly present with mild hypotonia and poor head control. The clinical presentation usually consists of muscle weakness and difficulty with ambulation. Patients will have difficulty moving up stairs, difficulty running, and are prone to falls. Weakness is typically more prominent in the proximal muscles of the lower limb [103]. Enlargement of the calves due to muscular atrophy and adipose replacement in pseudohypertrophy is seen classically in this condition. 

Confirmation of a diagnosis is typically performed through a muscle biopsy of the quadriceps femoris and gastrocnemius. On muscle biopsy, endomysial connective tissue proliferation, disorganized degeneration and regeneration of myofibers, and muscle fiber necrosis with a mononuclear infiltrate are expected findings. Muscle fiber necrosis will be present, involving characteristic replacement with adipose tissue as seen in pseudohypertrophy [103].

#### 3.1.3. Trichinosis

*Trichinella* are nematode parasites that infect humans, most commonly from the ingestion of larvae in undercooked, infected meat. They can be found in pigs, boars, and horses that have been infected from eating rats or food containing *T. spiralis*, *T. nativa*, *or T. britovi*. There are an estimated 10,000 cases around the world, but the incidence in the United States has dramatically decreased after the introduction of laws requiring the proper cooking of hog food. Once the larvae have been ingested, they develop into adults, begin to mate in the intestines, and then release more larvae. The larvae will travel systemically and invade muscle cells, causing a multitude of effects [106]. A muscle biopsy is the only way to prove absolute certainty of infection [107].

Infection with *Trichinella* can cause neurotrichinellosis which presents with encephalopathy along with neuromuscular and ocular disturbances [107]. Neurologic symptoms present in 10–20% of infected patients. Clinical presentation can include meningitis, encephalitis, paresis, and paralysis [108]. Imaging studies will show nodular multifocal hypodensities in patients who are serologically positive [107].

**Figure 3:**
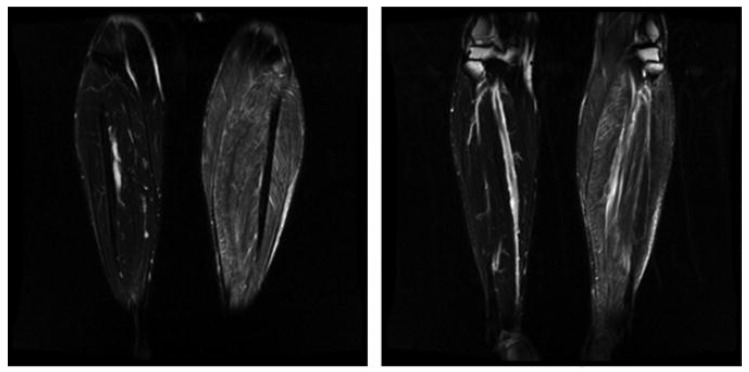
MRI of the lower limbs in the case of toxic myopathy. The T2 images show asymmetric involvement affecting only the **left** lower limb. Had a muscle biopsy been taken from the **right** gastrocnemius, the pathologic tissue would have been missed [4] © 2012 The Authors. Physical Medicine and Rehabilitation Clinics of North America by Elsevier.

Muscle biopsy is typically conducted in skeletal muscles, most commonly the diaphragm, extraocular, laryngeal, deltoid, gastrocnemius, and intercostal muscles (Figure 3). Histology will reveal larvae approximately 1 mm long surrounded by intracellular membrane-bound vacuoles. These membrane-bound vacuoles are surrounded by new blood vessels and an eosinophilic infiltrate. Infiltrates are most prominent around dying parasites, which result in calcification and scaring [106].

### 3.2. Muscle Biopsy: Low Indication

#### 3.2.1. Chloroquine Toxicity

Chloroquine is approved for use in the treatment of certain strains of malaria including *P. falciparum*, *P. ovale*, *P. vivax*, and *P. malariae*. Chloroquine works by preventing the polymerization of heme into hemozoin. This disruption in the metabolism of heme stops the malarial parasite from using hemozoin as a food source for proliferation [109]. Alternative indications for chloroquine are dermatomyositis, sarcoidosis, SLE, and some connective tissue diseases. Chloroquine can be used in these alternative diagnoses because of their modification of the immune system. Toxicity can affect cardiac and skeletal muscle, and muscle biopsy can be used as a diagnostic instrument to confirm the diagnosis [110].

Chloroquine toxicity can cause neuromyopathy that presents as a slowly progressing, painless proximal weakness. As toxicity progresses, patients experience muscle atrophy that is worse in the legs than arms. Toxicity can reduce sensation and stretch reflexes in muscles, most prominently at the ankles. In terms of dosing, neuromyopathy usually occurs in patients taking 500 mg/d for a year or greater but has been documented in patients taking doses as low as 200 mg/d. A study that tracked the prevalence of myopathy in patients taking antimalarials over the course of 3 years found that the incidence of myopathy was 9.2% [111]. Laboratory features of chloroquine toxicity will reveal elevated creatine kinase (CK) levels. In nerve conduction studies, a reduction in amplitude and reduced velocities can be observed [112].

Histology of a muscle biopsy will show autophagic vacuoles in 50% of skeletal and cardiac muscle, but type 1 fibers seem to be affected to a greater extent. The vacuoles seen will stain positive for acid phosphatase. When using electron microscopy, vacuoles contain concentric lamellar myeloid debris and curvilinear structures [110].

#### 3.2.2. Amiodarone Toxicity

Amiodarone is the most prescribed anti-arrhythmic medication in the United States [113]. It is indicated for the treatment of ventricular arrhythmias but is routinely used off-label to treat atrial fibrillation and to prevent ventricular tachyarrhythmias. Amiodarone is a class III anti-arrhythmic medication that blocks potassium currents that repolarize the myocardial cell in phase 3 of the action potential. Amiodarone also blocks beta 1 adrenergic receptors, calcium channels, and sodium channels. Therefore, it has properties of all four classes of anti-arrhythmic medications [113]. The diagnosis of toxicity can be confirmed by a muscle biopsy.

Amiodarone toxicity presents as a neuromyopathy with severe proximal and distal weakness. It also causes sensory loss and reduced stretch reflexes. Typically, the legs are more affected than the arms. The toxic effects are more significant in patients with hypothyroidism from amiodarone use and with renal insufficiency. It is important to note that the risk of myopathy is increased for patients who are also on a statin. Laboratory tests will show an elevated CK level and a nerve conduction study will show reduced amplitude and slowed conduction. These nerve conduction study (NCS) findings are especially prominent in the lower extremities. 

A muscle biopsy will reveal dispersed fibers with autophagic vacuoles. Neurogenic atrophy will be observed, especially in distal muscles. Electron microscopy will reveal myofibrillar disorganization and autophagic vacuoles that are filled with myeloid debris [111].

#### 3.2.3. Pompe Disease

Pompe disease is an inherited glycogen storage disease that causes a deficiency of lysosomal acid alpha-glucosidase [114,115]. Deficiency of this enzyme causes glycogen to deposit inside lysosomes within muscular tissue. The incidence of Pompe disease is approximately 1 in 40,000 in the US [115]. Although a muscle biopsy is not the only way to diagnose the condition, it can be used to recognize certain unusual variations in the disease. Muscle biopsy may play a particular role in the diagnosis of underreported variants that may be resistant to enzyme replacement therapy [114].

Pompe disease can present early in life as the infantile phenotype or later in life as the late-onset phenotype. The infantile phenotype presents with symptoms before the age of one. These symptoms include hypotonia, muscle weakness, motor delay, cardiomegaly, hepatomegaly, and respiratory failure. The late-onset phenotype exhibits symptoms in childhood or beyond. These include symptoms of proximal muscle weakness and respiratory failure. Progression in the late-onset phenotype is typically slower [115].

A muscle biopsy stained with hematoxylin and eosin will show glycogen-containing vacuoles that are nonspecific for glycogen storage diseases. The glycogen vacuoles will be in the lysosomes of muscle cells and will stain positively with a periodic acid–Shiff [114,116]. Lipofuscin inclusions can also be seen in patients with Pompe disease. These changes in muscle cells are absent in 20–30% of patients who have late-onset Pompe disease. Due to the low specificity of these findings, muscle biopsy is not the preferred method of diagnosis [114,115].

## 4. Emerging Biopsy Techniques

### 4.1. Image-Guided Biopsies

Using imaging modalities like computer tomography (CT), magnetic resonance neurography (MRN), or ultrasound (US) to guide the needle to the precise location has been shown to improve precision and accuracy, increase diagnostic yield, and reduce procedure time. The use of imaging also allows for smaller incisions, improved patient comfort, and decreased complication rates compared to traditional biopsy techniques.

The use of magnetic resonant imaging to visualize neurons, also called MRN, has seen a growing list of techniques, indications, and diagnostic applications in the past two decades [117,118]. MRNs depict the entire nerve in three dimensions, provide excellent soft tissue contrast, and function without operator skill limits. The soft tissue contrast allows for visualization of downstream muscle injury and high contrast resolution between surrounding fat and vascular structures, making it an excellent modality to guide nerve biopsy [6].

US excels in its provision of real-time visualization and comparison of nerves, portability, and low cost, while still allowing for dynamic imaging with high spatial resolution [119,120]. It furthermore operates without ionizing radiation and does not require contrast to operate. Conversely, it does come with certain limitations, chiefly the depth of range, soft tissue contrasts, and dependability on the operator’s skillset. Despite these limitations, developments in high-resolution US provide valuable neuro-metrics that serve to guide diagnostics and biopsy [121].

While CT scans have also historically been used to visualize nerves [122,123], US and MRN have emerged as the most far-reaching modalities for peripheral nerve imaging and often provide complimentary information [122,124,125]. The decision of which modality to use for a biopsy ultimately depends on the target nerve’s depth and accessibility, imaging availability, and the clinician’s preference. 

### 4.2. Optical Biopsy

Optical biopsy involves the use of specialized instruments that provide real-time microscopic images of tissues without the need to remove and process samples in a traditional manner. Techniques such as optical coherence tomography (OCT), confocal laser endomicroscopy, and fluorescence-guided biopsy have been explored in various settings for this purpose.

OCT uses low-coherence light to capture micrometer-resolution two- and three-dimensional images from within optical scattering media. Of note, this technique has been used in animal models to obtain high-resolution images of sciatic nerves [126,127], to monitor microvasculature flow around peripheral nerves as they are electrically stimulated [128,129], and to capture functional images of nerves [130]. The non-invasive nature of this technique makes biopsies of nerves possible without causing damage or impairing function, making studies such as those to visualize retinal nerve fibers in diabetic patients possible [131,132].

Another technique within the vein of optical biopsies is confocal microscopy. While outperformed by OCT in the depth of visualization and general clinical utility, this technique provides higher-quality images of superficial nerves. It has notably been used to assess peripheral neuropathy in vivo by visualizing epidermal nerves and corneal nerves [133,134,135]. While its application has seen a particular focus on small corneal nerve fibers to detect diabetic peripheral neuropathy [133,136,137], it may be potentially useful in other neuropathologies, such as Parkinson’s Disease [138], Friedreich’s ataxia [139], or amyotrophic lateral sclerosis [140].

**Figure 4:**
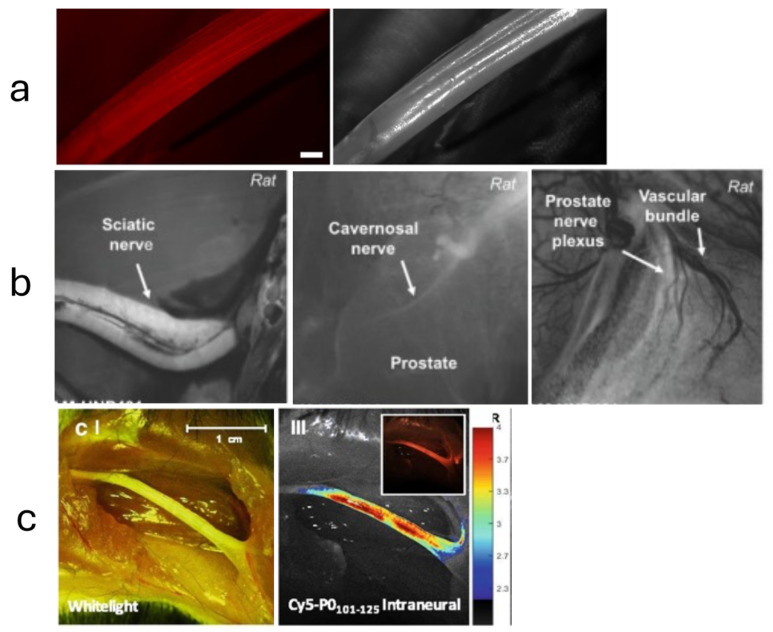
(**a**) Brightfield and corresponding fluorescence images of a mouse sciatic nerve taken at day six post intraperitoneal injection of a fluorescently labeled anti-ganglioside antibody, GT1b-2b-550. Adapted from the study by Massaad et al. [144] © 2015, Springer Nature. (**b**) Nerve-specific peptide FAM-HNP401 with an affinity for human nerve tissue showed an equivalent nerve-specific contrast in rats when compared to the previous FAM-NP41 nerve-specific peptide. Adapted from the study by Hingorani et al. [145] © 2018, Ivyspring International. (**c**) In vivo nerve specificity demonstration of a myelin protein zero (P0) derived peptide sequence, Cy5-P0101-125, following an intraneural injection at the site of a mouse sciatic nerve. Adapted from the study by Buckle et al. [146] © 2021, Springer. Caption adapted from the study by Wang & Gibbs. [146] © 2023, Elsevier.

A fluorescence-guided biopsy, which involves the use of nerve-specific fluorescent agents, can enhance the accuracy of tissue sampling and resection (Figure 4). While many studies focus on the use of fluorescent-guided imaging in the operative setting [125], the potential application to nerve biopsies is self-evident. One such technique targets Nav1.7 sodium channels, found in high density in peripheral nerves, with a selective fluorescent peptide. Using a surgical fluorescent microscope ex vivo, researchers were able to simulate a clinical scenario that identified the peripheral nerves via fluorescent highlights [141]. The growing success of fluorescence imaging has led many to seek out novel markers or strategies to visualize neurons in surgery or for biopsy [142,143].

### 4.3. Minimally Invasive and Target Fasicular Biopsy

Utilizing smaller instruments and techniques designed to reduce trauma, these biopsies aim to obtain tissue samples with minimal surgical intervention. Several studies have investigated how more superficial peroneal nerve and peroneus brevis muscle combined biopsies allow for the evaluation of both nerve and muscle tissues with high yield, sensitivity, and specificity for vasculitis neuropathies and other conditions [144,147]. The procedure’s minimal invasiveness allows for reduced morbidity compared to traditional sural nerve biopsy, highlighting a progression towards less invasive biopsy methods for nerve tissues without compromising diagnostic value.

Another approach, coined “targeted fascicular biopsy”, targets one or a few fascicles within a nerve, rather than taking a larger portion of the nerve. The goal of targeting smaller sensory and motor branches is to minimize the risk of worsening neurologic function secondary to biopsy, while still providing enough histopathological tissue to be diagnostic. This is accomplished by combining neuroimaging, clinical examination, and electrophysiological studies to identify the ideal nerve target, access the nerve as minimally as possible, and biopsy for pathological examination [145]. Selective and precise targeting allows for the biopsy of a redundant or expendable motor branch rather than resecting a fascicle from the parent nerve. This strategy has been applied to the biopsy of the brachial plexus [146] and the sciatic nerve and its major branches [146], among many others [145].

### 4.4. Shear Wave Elastography (SWE)

SWE is a diagnostic imaging modality that quantitatively assesses tissue stiffness by measuring the velocity of shear waves induced by an acoustic radiation force impulse. The propagation speed of these shear waves, which is directly proportional to tissue rigidity, provides clinicians with precise metrics of studied nerves [148]. SWE is gaining traction as a non-invasive technique to evaluate peripheral nerve integrity and pathology, potentially circumventing the need for invasive nerve biopsies.

Several studies are geared toward cataloging the elastic properties of healthy nerves for future reference and comparative assessment. This growing catalog currently includes the ulnar nerve [149], radial nerve [150], and median nerve [151], among many others. Other studies, such as one by Durand et al., 2021, have started comparing the elastic properties of healthy nerves against neuropathic nerves, in this case comparing entrapped ulnar nerves post decompression against the contralateral non-operative side [152].

Overall, SWE offers a non-invasive means to obtain quantitative data on tissue stiffness, potentially aiding in both the diagnosis and monitoring of various neuropathies while bypassing the risks of traditional surgical biopsy.

### 4.5. Small Fiber Diagnostic Approach and Needle Biopsies

Small Fiber Neuropathy (SFN) is characterized by damage to small, unmyelinated C fibers and thinly myelinated Aδ fibers. These fibers are crucial for pain and temperature sensation. Diagnosing SFN can be challenging as traditional nerve conduction studies often appear normal, warranting specialized diagnostic techniques to obtain tenable biopsies.

One of the primary diagnostic tools for SFN is skin biopsy, which involves taking small samples of skin to analyze the density of nerve fibers. The biopsy is typically taken from the distal leg and thigh, and the samples are stained and examined under a microscope to evaluate the density of intraepidermal nerve fibers (IENFs). A decreased IENF density confirms the diagnosis of SFN. This technique is minimally invasive and has become the gold standard for diagnosing SFN [153]. Additionally, quantitative sensory testing (QST) is used to assess the function of small nerve fibers by measuring the sensory threshold for various stimuli, such as temperature and pain. While not as definitive as a skin biopsy, QST provides valuable functional information about the small fibers [154].

Needle muscle biopsy is a less invasive alternative to open muscle biopsy and can be particularly useful in the diagnosis of certain neuromuscular diseases. This procedure involves inserting a hollow needle into the muscle to obtain a small tissue sample. Needle biopsies are typically performed under local anesthesia and can be performed in an outpatient setting.

Needle biopsies are effective in diagnosing conditions such as inflammatory myopathies, including polymyositis and dermatomyositis, as well as muscular dystrophies and other myopathies. The tissue obtained through a needle biopsy is processed and examined histologically, and can also be used for biochemical and molecular studies [155]. For increased accuracy and safety, ultrasound guidance, as previously mentioned, can be employed during needle biopsies [156]. Ultrasound allows for real-time visualization of the muscle, helping to precisely target the area of interest, which minimizes the risk of complications and increases the diagnostic yield.

In summary, the integration of small fiber diagnostic approaches and needle biopsies enhances the diagnostic landscape for neuromuscular diseases, providing valuable tools for clinicians to diagnose and manage these conditions effectively.

## 5. Conclusions

Neurosurgical intervention for nerve and muscle biopsies remains relevant in the current diagnostic landscape for many neurological and musculoskeletal disorders. Although biopsy may not be required for definitive diagnosis in certain conditions, the role of biopsy may still be essential when dealing with atypical cases and as a supportive point of evidence for diagnosis. In addition, several emerging techniques have been explored in the literature to guide diagnostics and biopsy, conduct less invasive biopsies, and reduce risks of worsening neurologic function and other symptoms secondary to biopsy.

## Figures and Tables

**Table 1:** Conditions in which nerve biopsies are of high, medium, and low importance.

High	Medium	Low
-Vasculitic Neuropathy	-Amyloidosis	-Chronic Inflammatory Demyelinating Polyneuropathy
-Neurolymphomatosis	-Neurosarcoidosis	-Paraproteinameic Neuropathy
-Peripheral Nerve Tumors		-Adult Polyglucosan Body Disease
-Pseudoneoplastic Peripheral Nerve Tumors	-IgG4-related Perineural Disease	-Lysosomal and Peroxisomal Storage Disorders
		-Pure Motor Neuropathy
-Neuritic Leprosy	-Paraneoplastic Syndromes	-Diabetic Neuropathy
		-Cryptogenic Neuropathy
		-Hereditary Neuropathy
		-Other Neuropathies

Source: Adapted from references [6,11,16].

**Table 2:** When biopsy is indicated for a suspected diagnosis.

Suspected Diagnosis	Case Where Biopsy Is Indicated
High Importance	
Vasculitic Neuropathy	Lack of evidence for extraneural vasculitis or progressive symptoms despite treatment
Neurolymphomatosis	Primary neurolymphomatosis or secondary neurolymphomatosis in cases of diagnostic ambiguity
Peripheral Nerve Tumors	Atypical benign tumors
Pseudoneoplastic Peripheral Nerve Tumors	Exclusion of malignancy
Neuritic Leprosy	Almost all cases for definitive diagnosis
Medium Importance	
Amyloidosis	Other tissue biopsy not possible or demonstrates negative results
Neurosarcoidosis	Negative results following extraneural biopsy or absence of extraneural symptoms
IgG4-Related Perineural Disease	Majority of patients, specifically in cases of atypical presentation or lack of extraneural evidence
Paraneoplastic Syndromes	In the setting of unclear etiologies of peripheral neuropathies
Low Importance	
Chronic Inflammatory Demyelinating Polyneuropathy	Lack of response to treatment or atypical presentation
Paraproteinameic Neuropathy	Suspected diagnosis of vasculitic or amyloid neuropathy, or infiltrative malignancy
Adult Polyglucosan Body Disease	Suspected diagnosis following inconclusive enzyme and genetic testing
Lysosomal and Peroxisomal Storage Disorders	Atypical presentation or suspected diagnosis following inconclusive testing
Pure Motor Neuropathy	Inability to determine etiology of neuropathy between motor neuron disease and motor neuropathy
Diabetic Neuropathy	Atypical presentation or suspected superimposed etiology
Cryptogenic Neuropathy	Suspected diagnosis following inconclusive testing
Hereditary Neuropathy	Atypical presentation or suspected diagnosis following inconclusive testing
Other Neuropathies	Atypical presentation or suspected diagnosis following inconclusive testing

Source: Adapted from references [6,11,16].
